# Characterization of a Diverse Collection of Salmonella Phages Isolated from Tennessee Wastewater

**DOI:** 10.1089/phage.2023.0004

**Published:** 2023-06-19

**Authors:** Daniel W. Bryan, Lauren K. Hudson, Jia Wang, Thomas G. Denes

**Affiliations:** Department of Food Science, University of Tennessee, Knoxville, Tennessee, USA.

**Keywords:** Salmonella phage, phage isolation, host range

## Abstract

**Background:**

*Salmonella enterica* is one of the most prevalent bacterial foodborne pathogens. *Salmonella* phages are currently used in biocontrol applications and have potential for use as therapeutics.

**Materials and Methods:**

Phages were enriched and purified from a diversity of *Salmonella* host isolates. Morphology was determined with transmission electron microscopy, host ranges were characterized using an efficiency of plaquing assay, and comparative genomic analysis was performed to determine taxonomy.

**Results:**

Ten phages were isolated and characterized. Phages showed activity against 23 out of the 24 *Salmonella* serovars evaluated. Two phages also showed activity against *Escherichia coli* strain B. Phages belonged to five different genera (*Ithacavirus*, *Gelderlandvirus*, *Kuttervirus*, *Tlsvirus*, and *Epseptimavirus*), two established species, and eight novel species.

**Conclusions:**

The phages described here further demonstrate the diversity of *S. enterica* phages present in wastewater effluent. This work contributes a collection of characterized phages from eastern Tennessee that may be of use in future phage-based applications targeting *S. enterica*.

## Introduction

Salmonellosis, caused by non-typhoidal *Salmonella* spp., is one of the most prevalent foodborne illnesses, with 58,371 reported cases (excluding *Salmonella* Typhi and *Salmonella* Paratyphi infection) in the United States in 2019.^1^ For FoodNet sites that same year, 29.0% of infections resulted in hospitalizations and 0.6% in deaths.^2^ In addition, there has been a concerning trend of increasing prevalence of antibiotic resistant isolates, including multi-drug resistant strains from both human outbreaks and in the food production environment.^3^

In light of this growing crisis of antibiotic resistance, there has been a concerted effort across industry, academia, and government to restrict the indiscriminate use of antibiotics and develop alternative antimicrobial agents for the control of pathogens in food production and processing environments.^4^

Bacteriophages are an alternative to conventional antimicrobials typically used in food production and processing environments. Currently, several commercial preparations of phage products targeting *Salmonella* spp. have been granted the GRAS (Generally Regarded As Safe) status by the Food and Drug Administration (FDA) and approved for use in multiple animal and food contexts.^5^ In addition to their ability to ignore chemical antibiotic resistance due to their different mechanism of action, the specificity of phages to their hosts means that phage treatments can target harmful bacteria without disrupting the broader microbiota.^6^

The specificity of bacteriophage host-recognition and their self-replicating capabilities give them unique advantages for applications in bacterial surveillance and diagnostics as well.^7^ However, this degree of host specificity necessitates diverse collections of well-characterized bacteriophages that are active against a broad range of target bacteria and that contain phages with both narrow and broad host ranges. These collections are an essential resource for developing effective phage-based applications^6^ and can be used as a base to develop specifically targeted phages using techniques such as biological engineering^8^ or directed evolution.^9^

In this study, we present a diverse collection of Salmonella bacteriophages isolated from wastewater effluent in East Tennessee, aiming at enabling their application in food safety and facilitating further research.

## Materials and Methods

### Bacterial strains and bacteriophages

All *Salmonella* strains in this study were previously used by Switt et al.,^10^ with the addition of *Escherichia coli* B to serve as an outgroup control, and to explore potential polyvalent activity against *Salmonella* and *E. coli*. All bacterial strains were stored at −80°C in tryptic soy broth (TSB) supplemented with 15% (v/v) glycerol. All bacterial liquid cultures were grown in TSB at 37°C in a shaking water bath at 160 RPM, and plate cultures were grown on tryptic soy agar (TSA) at 37°C. Overnight cultures were prepared from a single colony in 5 mL of TSB and incubated for 16 ± 2 h.

Bacteriophage enumeration was performed by serial dilution in pH 7.4 phosphate-buffered saline (PBS) followed by spot assay or double agar overlay. Briefly, spot assays were performed by adding 40 μL of overnight culture to 4 mL of molten TSA top agar held at 55°C (0.7% w/v agar), mixing by brief vortexing and immediately pouring the top agar on a 6 × 6 grid square plate of TSA. Then, 5 μL of selected serial dilutions were spotted onto the top agar lawn after it solidified before letting the spots dry and incubating at 37°C overnight.

Double agar overlay plates were prepared by adding 30 μL of overnight culture and 100 μL of a single phage dilution to 3 mL of molten TSA top agar held at 55°C, which were mixed by brief vortexing and immediately poured on a round petri dish of TSA agar before being incubated overnight at 37°C. Bacteriophage stocks were prepared in suspension medium (SM) buffer (0.1% v/v gelatin, 0.05 M tris-Cl pH 7.5, 0.58% w/v NaCl, 0.2% w/v MgSO_4_·7H_2_O).

Bacteriophage stocks were amplified by plate lysate. Briefly, several (4+ depending on desired concentration and volume) agar overlay plates of the selected phage were prepared at a dilution to produce near-confluent lysis of the bacterial lawn. After incubation overnight at 37°C, 5 mL of sterile SM buffer was aliquoted onto each plate and statically incubated at room temperature for 2 h. The buffer was then removed from the plate with a serological pipette, centrifuged at 5000 *g* for 20 min at 4°C to remove debris, and then filter sterilized using a 0.20 μm-pore size surfactant-free cellulose acetate (SFCA) syringe filter (Corning Incorporated, Corning, NY, USA). This filter sterilized stock was then concentrated by centrifugation at 12,000 *g* for 2 h, the supernatant was removed by a serological pipette, and the phage pellet was resuspended in sterile SM buffer by static incubation overnight at 4°C.

### Bacteriophage isolation

All bacteriophages described here were isolated from wastewater effluent collected after physical and primary treatment from the Kuwahee Water Treatment Plant in Knoxville, TN, USA. After collection, the effluent was centrifuged at 5000 *g* for 30 min, sterile filtered with a 0.45 μm SFCA membrane (Thermo Fisher Scientific, Waltham, MA, USA), and stored at 4°C. One hundred microliters of the effluent was then plated by double agar overlay with each strain and incubated at 37°C overnight; any well-defined and isolated plaques were picked from the plate with a 1 mL pipette tip and left to elute overnight in 1 mL of SM buffer with 5 μL CHCl_3_ for plaque purification.

An enrichment was performed for strains that did not produce plaques by direct plating of the effluent.^11^ Five milliliters of the effluent was added to 5 mL of 2 × concentration TSB and supplemented with CaCl_2_ to a final concentration of 2 mmol in an Erlenmeyer flask, 100 μL of overnight culture of the target strain was added, and the enrichment was incubated in a shaking water bath at 37°C for 24 h. After 24 h, 5 mL of the enrichment was removed; 1:200 CHCl_3_ was added, mixed, and statically incubated on the bench for 15 min before the enrichment was separated from the CHCl_3_, transferred to a 15 mL conical centrifuge tube, centrifuged at 5000 *g* for 20 min, and then filtered with a 1.2 μm filter followed by a 0.45 μm filter.

The filtered enrichment was then serially diluted in PBS and enumerated by double agar overlay. Individual, well-separated plaques from these plates were then collected. The remaining 5 mL of the enrichment was incubated for another 24 h and the remaining 5 mL was processed as previously described if no plaques were isolated from the 24 h sample.

Isolated plaques were plaque purified by serially diluting the plaque suspension and plating it by embedded agar overlay and then selecting and picking a single, well-separated plaque with a morphology consistent with the first plaque picked during isolation. This process was repeated a minimum of three times for every isolate until it exhibited a single, consistent plaque morphology via embedded agar overlay. After plaque purification, phage stocks were amplified by plate lysate as previously described. Ten phage isolates were selected for further characterization based on preliminary patterns of their ability to plaque on the 26 strains used in this study.

### Efficiency of plaquing assay

To determine the host ranges of these selected phages, an efficiency of plaquing (EOP) assay was performed as previously described^12^ but using TSB instead of LB-MOPS. Briefly, duplicate lawns of each strain were prepared for spot-assay plating, and 1 × 10^7^ PFU/mL working stocks of each phage were prepared and serially diluted in PBS to the 10^−5^ dilution. Five microliters aliquots of each dilution and the undiluted working stock were spotted on to the lawns, allowed to dry, and then incubated overnight at 37°C.

Three biological replicates were performed. Phage EOP was determined by averaging the results of the three replicates and using the highest dilution with countable plaques against a given strain relative to the number of plaques the phage was initially isolated on. Relative phage activity was not analyzed for this EOP, as phage activity was not observed in the absence of plaquing, which is typically seen for bacteriophages targeting the Gram-positive foodborne pathogen *Listeria monocytogenes*.^12,13^ Clustered heatmaps were generated via the pheatmap package in R.^14^

### Transmission electron microscopy

High titer (>1 × 10^10^ PFU/mL) phage stocks were prepared for transmission electron microscopy (TEM) as previously described with modifications.^15^ The phage stock was pelleted by centrifugation (21,000 *g* for 60 min) and washed with 0.1 M ammonium acetate solution (pH 7) three times before 10 μL of the washed stock was deposited on a 200 mesh carbon-coated Formvar film copper grid (Electron Microscopy Sciences, Hatfield, PA, USA) and stained with UranyLess EM Stain (Electron Microscopy Sciences) before imaging on a JEOL JEM 1400-Flash transmission electron microscope (JEOL, Akishima, Tokyo, Japan) at 120 kV. Images were analyzed using Fiji v. 2.9.0,^16^ and TEM figures were prepared using the FigureJ plugin.^17^

### DNA extraction, sequencing, and genomic analysis

Phage DNA was extracted from purified stocks by phenol-chloroform extraction as previously described^15^ with additional quantification of DNA concentration by a Qubit 4 Fluorometer (Invitrogen, Waltham, MA, USA). The phage DNA was shipped to the SeqCenter (Pittsburgh, PA, USA) for sequencing. Sample libraries were prepared using the Illumina DNA Prep kit and IDT 10 bp UDI indices (Illumina, San Diego, CA, USA), and it was sequenced on an Illumina NextSeq 2000, producing 2 × 151 bp reads. Demultiplexing, quality control, and adapter trimming were performed with bcl-convert v3.9.3 (Illumina, 2021).

The initial bioinformatic analysis was performed on the KBase online platform.^18^ Quality control of the raw sequence reads was performed using FastQC^19^ (v0.11.9). The reads were then trimmed using Trimmomatic^20^ (v0.36; with the following parameters: Leading 3, Trailing 3, SlidingWindow 4:15, MinLen 36). Genome assembly was carried out using Unicycler^21^ (v0.4.8) with default settings. Phage assemblies were reoriented manually using Geneious Prime (version 2022.2.2) according to the guidelines in Shen and Millard.^22^

The reoriented assemblies were annotated using RASTtk^23^ (v1.073). The annotation of antibiotic resistance genes was performed by ResFinder^24^ (version 4.1) available on the Center for Genomic Epidemiology website. The virulence factor database^25^ (VFDB) was used for the predictions of toxin-encoding genes in phage genomes. The lifestyles of phages were predicted using PhageAI^26^ (version 1.0.2). The completeness and contamination of the phage genomes were assessed using CheckV^27^ (version 0.0.1).

BLAST^28^ was used to determine the most closely related genera for each phage. For each genus, assemblies of International Committee on Taxonomy of Viruses (ICTV) species exemplars and unclassified isolates were downloaded from NCBI Refseq or GenBank, respectively. VICTOR^29^ and VIRIDIC^30^ (web version available at viridic.icbm.de; default settings) were used to determine genome-based phylogeny and taxonomic classification. All raw sequencing reads and genome assemblies were submitted to NCBI under BioProject PRJNA915379; GenBank accessions are listed in Table 2.

## Results

### Morphological determination by TEM

**FIG. 1.**
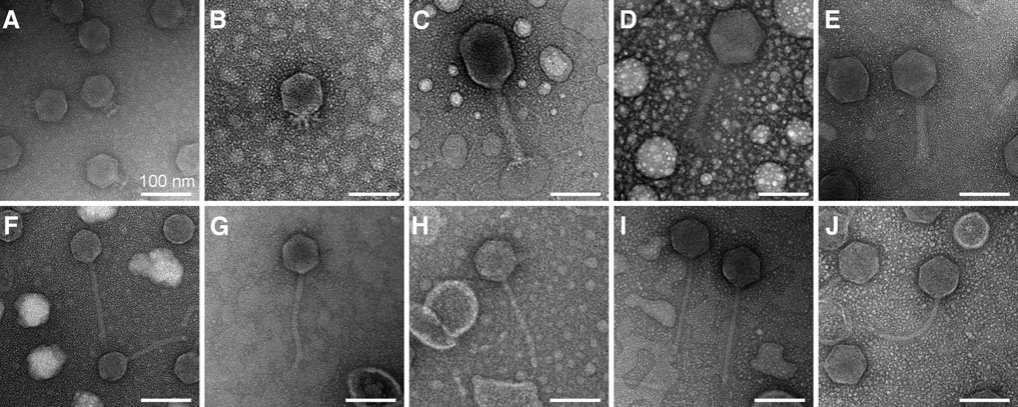
Transmission electron micrographs of bacteriophages. Bacteriophages as imaged by TEM. **(A)** UTK0001 (podovirus), **(B)** UTK0002 (podovirus), **(C)** UTK0003 (myovirus), **(D)** UTK0004 (myovirus) **(E)** UTK0005 (myovirus), **(F)** UTK0006 (siphovirus), **(G)** UTK0007 (siphovirus) **(H)** UTK0008 (siphovirus), **(I)** UTK0009 (siphovirus), **(J)** UTK0010 (siphovirus). All samples were strained with UranyLess EM Stain. All scale bars are 100 nm in length. TEM, transmission electron microscopy.

The morphology of these phages was observed by TEM (Fig. 1). Based on these images, UTK0001 and UTK0002 display the short, non-contractile tail characteristic of podoviruses; UTK0003, UTK0004, and UTK0005 exhibit the long, rigid, contractile tails of myoviruses (with UTK0003 exhibiting a slightly elongated capsid compared to the other myoviruses), and the remaining phages (UTK0006-UTK0010) have the long, flexible, non-contractile tails of siphoviruses (Table 1).

**Table 1.** The 10 Phages Isolated for Downstream Genomic Analysis

Formal name	Isolation host (serovar)	Morphology	Family (subfamily)	Genus or species
vB_SenP_UTK0001	FSL A4-0525 (Anatum)	Podovirus	*Schitoviridae (Humphriesvirinae)*	*Ithacavirus* ^a^
vB_SenP_UTK0002	FSL S5-0464 (Stanley)	Podovirus	*Schitoviridae (Humphriesvirinae)*	*Ithacavirus* ^a^
vB_SenM_UTK0003	FSL S5-0506 (Infantis)	Myovirus	*Straboviridae (Tevenvirinae)*	*Gelderlandvirus s16*
vB_SenM_UTK0004	FSL S5-0455 (Heidelberg)	Myovirus	*Ackermannviridae (Cvivirinae)*	*Kuttervirus* ^a^
vB_SenM_UTK0005	FSL S5-0370 (Typhimurium)	Myovirus	*Ackermannviridae (Cvivirinae)*	*Kuttervirus* ^a^
vB_SenS_UTK0006	FSL S5-0406 (Javiana)	Siphovirus	*Drexlerviridae (Tempevirinae)*	*Tlsvirus* ^a^
vB_SenS_UTK0007	FSL S5-0369 (Saintpaul)	Siphovirus	*Drexlerviridae (Tempevirinae)*	*Tlsvirus* ^a^
vB_SenS_UTK0008	FSL R8-0092 (Corvallis)	Siphovirus	*Demerecviridae (Markadamsvirinae)*	*Epseptimavirus LVR16A*
vB_SenS_UTK0009	FSL S5-0373 (Braenderup)	Siphovirus	*Demerecviridae (Markadamsvirinae)*	*Epseptimavirus* ^a^
vB_SenS_UTK0010	FSL R8-0376 (Oranienburg)	Siphovirus	*Demerecviridae (Markadamsvirinae)*	*Epseptimavirus* ^a^

^a^ Novel species.

### Genome sequencing and analysis of the phages

The paired end reads from the Illumina sequencing of the 10 phages were used to perform the *de novo* assembly with Unicycler, and a circular genome was generated for each phage. The reconstructed phage genomes ranged from 50 to 160 kbp in length (Table 2), with numbers of coding sequences (CDSs) ranging from 85 to 261 and GC content between 36.88% and 45.10%.

**Table 2.** Phage Genome Statistics

Name Accession	Length (bp)	GC content (%)	No. CDS	No. hypothetical protein	No. tRNA	Closest phage (accession)	Genomic similarity (%)
UTK0001 OQ359891 GCA_028897605.1	71,436	39.78	93	14	11	Shigella phage B2 (GCA_024606155.1)	92.905
UTK0002 OQ359892 GCA_028897615.1	70,977	39.7	89	15	11	Salmonella phage FSL SP-058 (GCF_000908895.1)	93.933
UTK0003 OQ359887 GCA_028897565.1	160,221	36.88	261	35	3	Salmonella phage vB_SenM-S16 (GCF_000905195.1)	100.0
UTK0004 OQ359883 GCA_028897525.1	157,790	45.1	205	29	5	Salmonella phage vB_SenA_SilasIsHot (GCA_016455765.1)	93.401
UTK0005 OQ359884 GCA_028897535.1	157,767	44.7	207	27	6	Salmonella phage vB_SenA_Guerrero (GCA_025887965.1)	93.421
UTK0006 OQ359885 GCA_028897545.1	49,758	42.8	85	11	0	Citrobacter phage_vB_CfrD_Devorator (GCA_021355995.1)	89.820
UTK0007 OQ359886 GCA_028897555.1	51,921	43.17	92	11	0	Salmonella phage vB_SenS_PHB07 (GCF_003031035.1)	89.456
UTK0008 OQ359888 GCA_028897575.1	111,604	40.08	159	5	23	Salmonella phage LVR16A (GCF_003328985.1)	99.999
UTK0009 OQ359889 GCA_028897585.1	113,742	40.3	161	4	24	Salmonella phage Seabear (GCA_005574435.1)	82.036
UTK0010 OQ359890 GCA_028897595.1	113,248	40.29	162	7	24	Phage A148 (GCA_002956865.1)	92.421

Genome assemblies are available at https://www.ncbi.nlm.nih.gov/bioproject/PRJNA915379.

The completeness of all genomes was determined by CheckV to be no <96% without any contamination. No tRNA CDSs were detected in the genomes of phage vB_SenS_UTK0006 and vB_SenS_UTK0007. The genome analysis of the 10 phages showed that all their genomes contained genes encoding proteins involved in DNA replication, metabolism, phage packaging, and structure. The genomes of phage UTK0003–UTK0010 also contained genes coding for cell lysis proteins. No antibiotic resistance, toxin or lysogeny-related genes were detected in the genomes of 10 phages.

**FIG. 2.**
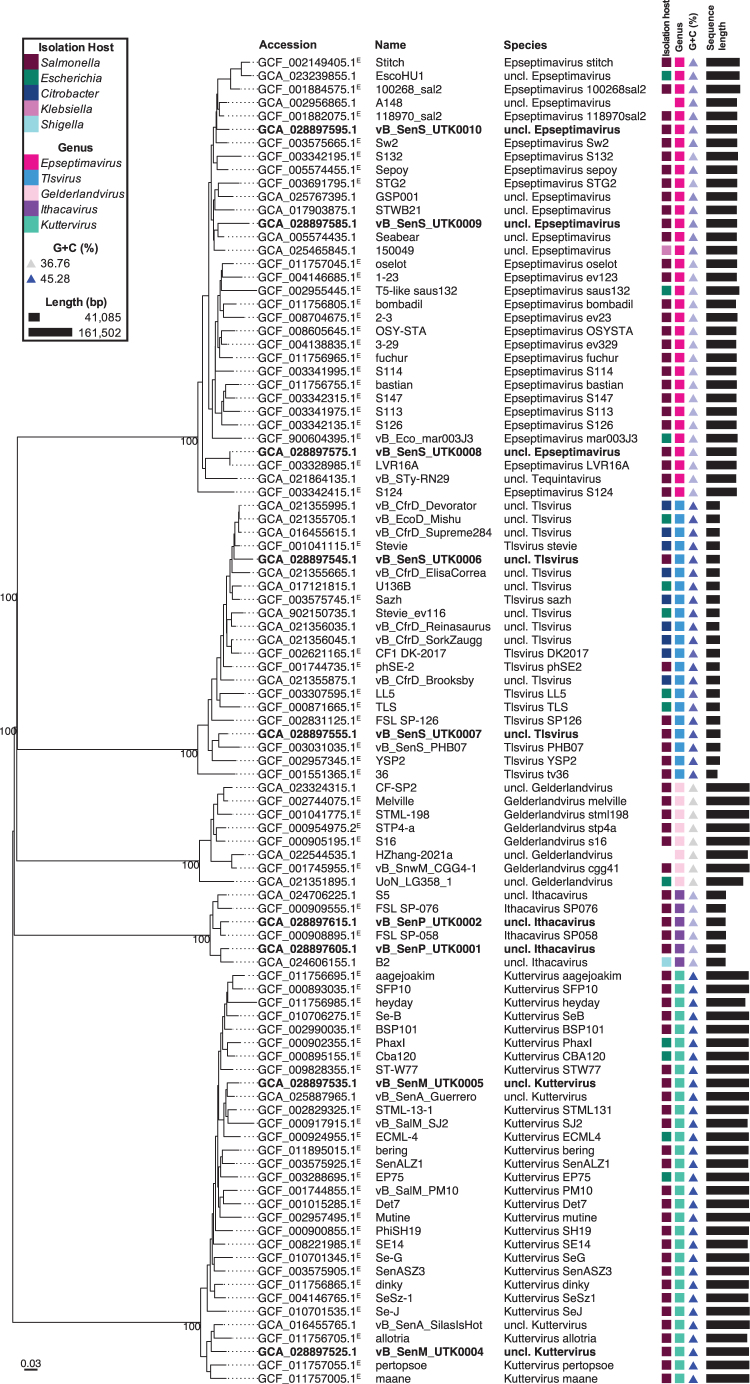
Phylogenomic tree with taxonomic classifications. Tree was created using intergenomic distances to infer a minimum evolution tree with VICTOR. Distances were calculated based on distance formula *d*_0_ (length of all HSPs divided by total genome length), and branch lengths were scaled in terms of this distance formula. The numbers above branches are GBDP pseudo-bootstrap support values from 100 replications. Labels contents (from left to right): accession (ICTV species exemplars are followed by a superscript “E”), phage name, species, isolation host, genus, G + C content (%; blue triangles, lighter shade corresponds to lower values and darker shade to higher values), and sequence length (black bars, bar length corresponds to genome length). Labels for phages from this study are in bold. UTK0003 was not included, because VICTOR determined that it was a duplicate of GCF_000905195.1. GBDP, Genome-BLAST Distance Phylogeny; HSP, high-scoring segment pair; ICTV, International Committee on Taxonomy of Viruses.

Two different programs (VICTOR and VIRIDIC) were used to evaluate genome-based phylogeny and taxonomy, with consistent results (Fig. 2; Supplementary Fig. S1). The 10 phages belong to 5 different families (*Schitoviridae*, *Straboviridae*, *Ackermannviridae*, *Drexlerviridae*, and *Demerecviridae*) and five different genera (*Ithacavirus*, *Gelderlandvirus*, *Kuttervirus*, *Tlsvirus*, and *Epseptimavirus*) (Table 1 and Fig. 2; Supplementary Fig. S1). Based on an intergenomic similarity (as determined by VIRIDIC) threshold of 95% for species demarcation, eight belong to novel species and two belong to established species.

The Ithacaviruses, UTK0001 and UTK0002, were most closely related to Shigella phage B2 and Salmonella phage FSL SP-058 (ICTV species exemplar), respectively (Table 2). Both assemblies were re-oriented to maintain consistency with FSL SP-058. Based on intergenomic similarity (as determined by VIRIDIC), both isolates represent novel species within the *Ithacavirus* genus (Table 2; Supplementary Fig. S1). Ithacaviruses belong to the family *Schitoviridae*, which are characterized by podovirus morphology and 59–80 kb linear genomes with defined ends (terminal repeats expected).^31^

The *Gelderlandvirus*, UTK0003, was most closely related to Salmonella phage S16 (ICTV species exemplar) (Table 2), and the assembly was re-oriented to start at the RIIA gene to maintain consistency with previously submitted genomes. Based on intergenomic similarity, UTK0003 is indistinguishable (100.0% similarity) from Salmonella phage vB_SenM-S16 and would belong to the *Gelderlandvirus s16* species (Table 2; Supplementary Fig. S1). Previously characterized *Gelderlandvirus* genomes have been described as circularly permuted, linear dsDNA.^32^

The Kutterviruses, UTK0004 and UTK0005, were most closely related to Salmonella phage vB_SenA_SilasIsHot and Salmonella phage vB_SenA_Guerrero, respectively (Table 2). These genomes were re-opened to start at the rIIA gene to maintain consistency with previously submitted genomes. Based on intergenomic similarity, UTK0004 and UTK0005 each represent a novel species within the *Kuttervirus* genus (Table 2; Supplementary Fig. S1). Previously characterized *Kuttervirus* genomes have been described as terminally redundant and circularly permuted.^33,34^

The Tlsviruses, UTK0006 and UTK0007, were most closely related to Citrobacter phage_vB_CfrD_Devorator and Salmonella phage vB_SenS_PHB07, respectively (Table 2). These assemblies were re-oriented to maintain consistency with previously submitted genomes. Based on intergenomic similarity, UTK0006 and UTK0007 each represent a novel species within the *Tlsvirus* genus (Table 2; Supplementary Fig. S1).

The Epseptimaviruses, UTK0008, UTK0009, and UTK0010, were most closely related to Salmonella phage LVR16A, Salmonella phage Seabear, and phage A148, respectively (Table 2). These assemblies were re-oriented to maintain consistency with previously submitted genomes. Based on intergenomic similarity, UTK0008 is very closely related to Salmonella phage LVR16A (99.999%) and would belong to the *Epseptimavirus LVR16A* species (Table 2; Supplementary Fig. S1).

UTK0009 and UTK0010 each represent a novel species within the *Epseptimavirus* genus. Epseptimaviruses belong to the family *Demerecviridae*, which are characterized by 106–123 kb genomes,^35^ which is consistent with the genome lengths of these three assemblies. Previously characterized Epseptimaviruses have been described as having linear dsDNA genomes, with 9–11 kbp direct terminal repeats.^36–38^

### EOP assay

**FIG. 3.**
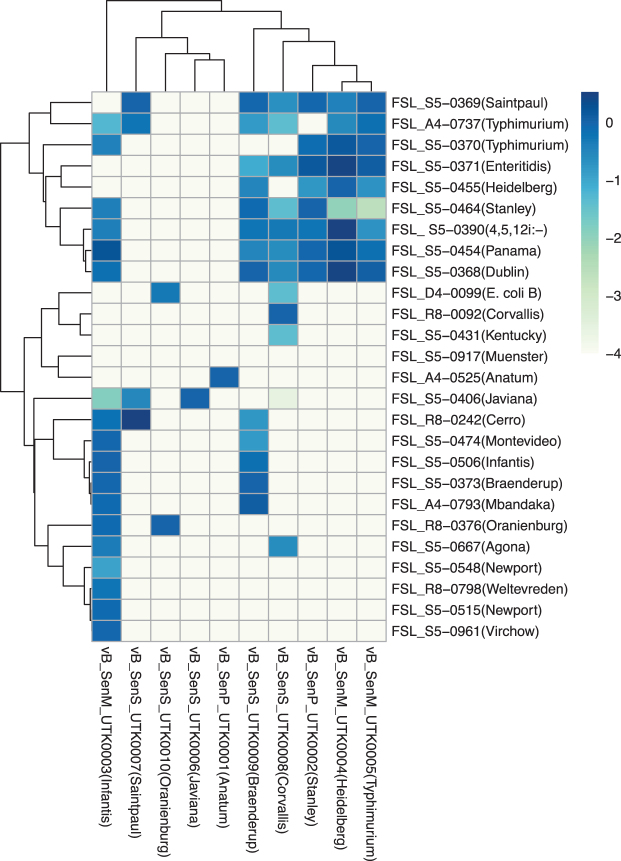
EOP heatmap. Heatmap indicates the mean EOP of Salmonella phages on a panel of *Salmonella enterica* strains and one strain of *Escherichia coli* (*n* = 3). Values are the log-transformed efficiencies of plaquing of phages against each strain relative to that against their isolation strain. EOP, efficiency of plaquing.

To assess the host range of these phages, an EOP assay was performed using 25 *Salmonella* strains, which represent a diverse collection of *Salmonella enterica* serovars isolated from human and bovine sources,^10^ and the lab strain *E. coli* B. The results of this assay (Fig. 3) show a great diversity of host ranges, with three phages with relatively broad host ranges (plaquing on 10 or more strains), three phages with a relatively middling host ranges (plaquing on five to nine strains), and three phages with relatively narrow host ranges (<4 strains).

Of the phages with narrow host ranges, two (UTK0001 and UTK0006) formed plaques only on the strain they were isolated from, and one phage (UTK0010; isolated from serovar Oranienburg) was capable of forming plaques on its isolation strain and *E. coli* B (one of the two phages that formed plaques on *E. coli*). Only UTK0004 and UTK0005 (isolated from serovar Heidelberg and Typhimurium respectively, but both in the *Kuttervirus* genus) formed plaques on the same strains, but with different efficiencies.

The other phages that share a genus do not exhibit such similar plaquing patterns. Among the 26 strains tested, only one strain was resistant to every phage tested (FSL S5-0917 serovar Muenster) and three strains were infected by a single phage (serovar Anatum, Kentucky, Corvallis, Newport, Weltevreden, and Virchow). Among the phages that formed plaques on the strains infected by a single phage, only UTK0003 and UTK0008 showed relatively broad host ranges against the other strains from this study.

## Discussion

To expand our knowledge of the diversity of bacteriophages of *S. enterica*, we report the isolation and characterization of 10 *S. enterica* bacteriophages isolated from wastewater effluent in eastern Tennessee. Wastewater effluent is a well-established source of bacteriophages^39^ for many organisms, including *S. enterica*,^40–42^ and is suggested to offer a better reservoir for isolating phages that have a broader host range than phages isolated directly from feces or fecal storage on farms that have been shown to contain more phages with restricted host ranges.^43,44^ This study further demonstrates that even a single sample of municipal wastewater offers an abundant and diverse source of novel *S. enterica* phages and contributes phages isolated from eastern Tennessee to the regional diversity of characterized *Salmonella* bacteriophages.

The 10 phages presented in this article represent 5 genera and the 3 major bacteriophage morphotypes (3 myoviruses, 2 podoviruses, and 5 siphoviruses). Eight of the phages belong to novel species, and two belong to established species. No genes coding for virulence, antibiotic resistance, or lysogeny were predicted in the 10 phage genomes, suggesting that there will be no unintended effects on the host bacteria when applied—though further testing would be needed to verify the absence of such genes before using these phages in an applied context.

These phages also demonstrate a diversity of host ranges as determined by EOP, ranging from UTK0003, a *Gelderlandvirus* myovirus isolated from *Salmonella* Infantis, which formed plaques on 18 out of 26 of the strains tested (including four strains that were resistant to all other phages described here), to UTK0006, a *Tlsvirus* siphovirus that only forms plaques on its isolation host *Salmonella* Javiana. While phages with as broad host ranges as possible are generally preferred for biocontrol or therapeutic applications, phages with more restricted host ranges remain of value for targeted interventions or phage-based detection or diagnostic applications.^45^

Three of the phages characterized here, UTK0001, UTK0008, and UTK0010 showed particularly interesting phenotypes. UTK0001, a podovirus in the genus *Ithacavirus*, is specific to its isolation host *Salmonella* Anatum (FSL A4-0525) and is the only phage in this collection that forms plaques on the said strain. UTK0008 and UTK0010, both siphoviruses in the *Epseptimavirus* genus, are the only phages in this collection that were capable of forming plaques on the laboratory strain *E. coli* B.

UTK0008 formed plaques on 12 of the 26 tested strains, making it similar to several previously reported polyvalent phages isolated from *Salmonella* that are capable of infecting both *S. enterica* and *E. coli*. These polyvalent phages have exhibited broad host ranges within *Salmonella*, in addition to forming plaques on *E. coli*.^46–48^ However, the other phage that can infect *E. coli* B, UTK0010, was only able to form plaques on its isolation host *Salmonella* Oranienburg (FSL R8-0376).

Interestingly, some phages polyvalent for *E. coli* and *S. enterica* isolated on *E. coli* appear to exhibit broad host ranges on *E. coli* and limited activity on *S. enterica*;^49^ future work can further explore UTK0010's host range with a larger collection of *S. enterica* and *E. coli* strains to better characterize its polyvalent activity and ability to infect an even more diverse selection of *S. enterica* strains.

This work contributes a diverse collection of characterized phages that were isolated from eastern Tennessee that may be of use in future phage-based applications targeting *S. enterica*. The diversity of phages described here further demonstrates that wastewater effluent is an abundant source for the isolation of novel *S. enterica* phages.

## Supplementary Material

Supplemental data
