# Supplementary material for: Characterization of a Diverse Collection of Salmonella Phages Isolated from Tennessee Wastewater
Source: Phage (New Rochelle). 2023 Jun 19;4(2):90–8. doi: 10.1089/phage.2023.0004 (PMC10282790; doi:10.1089/phage.2023.0004)
Supplement: Supplemental data [file Suppl_FigureS1.docx]

**Supplemental Figure 1.** Intergenomic similarities heatmap

Heatmap was created using VIRIDIC by computing pairwise intergenomic similarities (blue) and clustering using the complete clustering method. The alignment fraction (green) and length (purple) ratios for each pair are also indicated. Only values over 50% similarity and 0.5 ratios are shown.
